# Combined evaluation of glomerular phospholipase A2 receptor and immunoglobulin G subclass in membranous nephropathy

**DOI:** 10.1093/ckj/sfae104

**Published:** 2024-04-17

**Authors:** Kenji Ueki, Akihiro Tsuchimoto, Yuta Matsukuma, Eri Ataka, Hirofumi Okamoto, Shigeru Tanaka, Kosuke Masutani, Takanari Kitazono, Toshiaki Nakano

**Affiliations:** Department of Medicine and Clinical Science, Kyushu University, Fukuoka, Japan; Department of Medicine and Clinical Science, Kyushu University, Fukuoka, Japan; Department of Medicine and Clinical Science, Kyushu University, Fukuoka, Japan; Department of Medicine and Clinical Science, Kyushu University, Fukuoka, Japan; Department of Medicine and Clinical Science, Kyushu University, Fukuoka, Japan; Department of Medicine and Clinical Science, Kyushu University, Fukuoka, Japan; Division of Nephrology and Rheumatology, Department of Internal Medicine, Faculty of Medicine, Fukuoka University, Fukuoka, Japan; Department of Medicine and Clinical Science, Kyushu University, Fukuoka, Japan; Department of Medicine and Clinical Science, Kyushu University, Fukuoka, Japan

**Keywords:** complement, immunoglobulin G subclass, membranous nephropathy, oligosaccharide, phospholipase A2 receptor

## Abstract

**Background:**

Phospholipase A2 receptor (PLA2R) is a major target antigen in idiopathic membranous nephropathy (MN). Anti-PLA2R antibodies are mainly of the immunoglobulin G (IgG) subclass IgG4, although other IgG subclass depositions in glomeruli may also be detected. However, the importance of the subclass of the IgG deposit has not been proven. Thus we investigated clinical findings from patients with idiopathic MN in relation to glomerular PLA2R deposition and IgG subclass.

**Methods:**

We enrolled 132 Japanese patients with biopsy-proven idiopathic MN in a multicentre retrospective observational study. We investigated the complete remission rate as the primary outcome and the development of end-stage kidney disease (ESKD) as the secondary outcome in relation to glomerular PLA2R deposition. Moreover, we evaluated prognostic factors, including glomerular IgG subclass, in the PLA2R-positive group.

**Results:**

The percentage of cases with glomerular PLA2R deposition was 76.5% (*n* = 101). The first complete remission rate of the PLA2R-positive group was worse than that of the PLA2R-negative group (logrank test *P* < .001). ESKD incidence did not significantly differ between the glomerular PLA2R-negative and PLA2R-positive MN groups (logrank test *P* = .608). In the PLA2R-positive group, higher PLA2R intensities and IgG2 staining were associated with a poorer first complete remission rate (logrank test *P* < .001 and *P* = .032, respectively). Cox proportional hazards analysis also showed that strong PLA2R deposition and positive IgG2 staining were significantly associated with a failure to reach complete remission [hazard ratio 2.09 (*P* = .004) and 1.78 (*P* = .030), respectively].

**Conclusions:**

Our results suggest that intense glomerular PLA2R and IgG2 positivity predict a poor proteinuria remission rate in idiopathic MN.

## Graphical Abstract

**Graphical Abstract:**
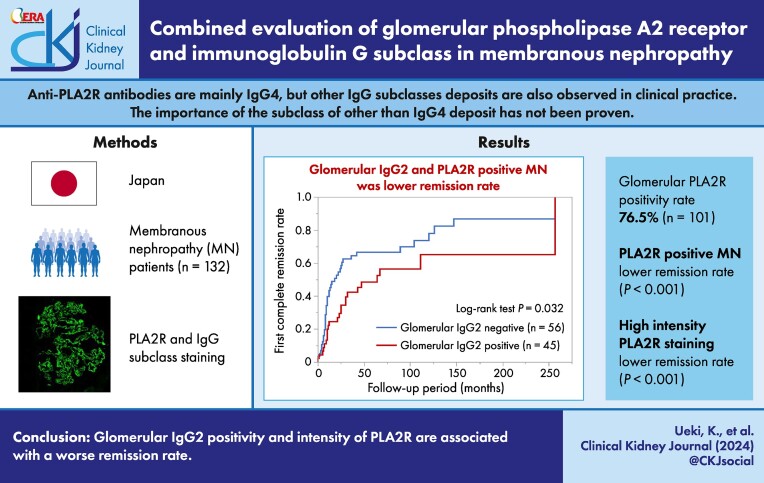

KEY LEARNING POINTS **What was known:**

- Phospholipase A2 receptor (PLA2R) is a major antigen in idiopathic membranous nephropathy (MN).
- The main immunoglobulin G (IgG) subclass of anti-PLA2R antibodies is IgG4, although glomerular deposits of other IgG subclasses are also occasionally observed.
- The importance of deposits of subclasses other than IgG4 has not been proven.

**This study adds:**

- This report revealed that the complete remission rate of the PLA2R-positive group was worse than that of the PLA2R-negative group.
- In the PLA2R-positive group, the intensity of PLA2R staining and IgG2 positivity predicted a poor proteinuria complete remission rate.

**Potential impact:**

- This report demonstrated the importance of combined analysis of PLA2R staining intensity and IgG2 positivity, which can predict disease activity.
- IgG2, which is associated with oligosaccharides, may cause significant pathogenesis in MN via epitope spreading or complement pathway activation through the lectin pathway.

## INTRODUCTION

Membranous nephropathy (MN) is one cause of nephrotic syndrome in adults. Histologically, MN is characterized by the deposition of autoimmune complexes on the subepithelial side of the glomerular basement membrane (GBM). The autoimmune complexes can be observed as spike formations by light microscopy and immunoglobulin G (IgG) deposition by immunofluorescence (IF) microscopy. The prognosis for patients with MN varies [1]. Some cases can reach spontaneous remission without special treatment, but others progress to end-stage kidney disease (ESKD) even with strong immunosuppressive therapy with glucocorticoids or immunosuppressants. Therefore, the identification of prognostic factors for MN is essential to improve clinical practice and patient outcomes.

Phospholipase A2 receptor (PLA2R) is the most clinically important target antigen in MN [2]. The serum positivity rate of patients with idiopathic MN for anti-PLA2R antibodies is reported to be around 70% [3, 4]. Moreover, because the titre of serum anti-PLA2R antibodies is associated with disease activity [5], it is useful for predicting treatment response. However, serum antibody titres may be negative in patients with weak or early-stage disease [6]. In addition, a recent meta-analysis revealed that the sensitivity of serum antibodies against PLA2R is relatively low at 69%, despite their high specificity of 99% [3]. Although glomerular PLA2R antigen can be detected with high sensitivity in renal biopsies by IF staining [6, 7], the clinical relevance of this staining has not been adequately investigated.

Glomerular IgG subclass staining has been used to distinguish between secondary MN, such as lupus nephritis, and idiopathic MN [8–10]. Most anti-PLA2R antibodies are of the IgG subclass IgG4 [11], but glomerular staining for the other IgG subclasses (IgG1–3) is occasionally observed in clinical settings in PLA2R-positive patients with MN. Although many previous reports have examined the whole pattern of staining of IgG subclasses, few previous reports have examined the individual staining findings for a subclass other than IgG4. The clinical importance of IgG subclass remains to be fully investigated.

We investigated the remission rate of patients with idiopathic MN based on glomerular PLA2R deposition and the relationship between glomerular PLA2R and IgG subclass in renal biopsy samples.

## MATERIALS AND METHODS

### Study design and population

This was a multicentre retrospective observational study. From January 1998 to December 2018, a total of 250 patients were diagnosed with MN by renal biopsy at Kyushu University Hospital, Karatsu Red Cross Hospital, Kyushu Central Hospital, Hamanomachi Hospital, Hakujyuji Hospital and Munakata Medical Association Hospital in Japan. Of these, 80 were excluded for practical reasons, including 51 who had no frozen biopsy sample to stain for PLA2R, 10 with medical charts that were too old to access, 15 who were lost to follow-up within 6 months of renal biopsy and 4 who were later diagnosed as not MN. A further 38 considered to be secondary MN were excluded for the following reasons: 7 were diagnosed with lupus nephritis, 10 with MN induced by antirheumatic drugs such as bucillamine, 3 with graft-versus-host disease after hematopoietic stem cell transplantation, 2 with antineutrophil cytoplasmic antibody–associated vasculitis, 4 with IgG4-related kidney disease, 1 with amyloidosis, 1 with Cronkhite–Canada disease and 6 with hepatitis B or C virus infection. Although it is difficult to determine if the cause of MN is malignancy, based on previous reports [12] we excluded four cases with a diagnosis of malignancy within 1 year before or after the onset of MN or with active malignancy at the time of MN diagnosis by renal biopsy. The remaining 132 patients were enrolled in accordance with a registered study protocol (Supplementary Fig. S1).

The present observational study was performed in accordance with the guidelines of the Declaration of Helsinki. The Japanese hospitals that enrolled patients in this multicentre study participate in the Fukuoka Kidney Disease Registry Study [13]. The study was registered at the University Hospital Medical Information Network clinical trial registry (UMIN000007988) and was approved by the institutional review boards of the Human Ethics Committee of Kyushu University Hospital (approval 469-09).

### Clinical measurements and definitions

Demographic and clinical data were retrospectively obtained from medical records. Baseline characteristics at the time of renal biopsy, duration from onset to renal biopsy, history of malignancy, details of treatment after renal biopsy, kidney prognosis and complete and partial remission were obtained. The clinical parameters included sex, age, systolic blood pressure (BP), diastolic BP, haematuria, urinary protein:creatinine ratio (UPCR) and levels of serum total protein, serum albumin, blood urea nitrogen, serum creatinine, serum total cholesterol, serum triglyceride and serum IgG.

Haematuria was defined as >5 red blood cells/high-power field in the urine sediment test. Nephrotic syndrome was defined as urinary protein >3.5 g/g creatinine and a serum albumin level <3.0 g/dl.

The pathological parameters measured by light microscopy included glomerular IgG subclass staining, spike formation of the GBM, GBM thickness, GBM double contour, interstitial fibrosis and tubular atrophy (IFTA) and interstitial cell infiltration. The diagnosis of MN was based on the pathological parameters of GBM thickness or double contour, spike formation as observed after periodic acid–methenamine silver staining and glomerular IgG deposition as assessed by IF staining. Glomerular IgG subclass stains were performed on frozen sections using fluorescein isothiocyanate-conjugated antibodies (IgG1: AF006, IgG2: AF007, IgG3: AF008 and IgG4: AF009; all antibodies were supplied by The Binding Site, Birmingham, UK). IFTA was quantified and interstitial cell infiltration defined based on the ratio of lesion area to cortex area (1+: 10–24%, 2+: 25–49%, and 3+: >50%).

For treatment, the Kidney Disease: Improving Global Outcomes (KDIGO) guidelines [14] recommend risk classification, which may require >6 months of follow-up with conservative treatment. We obtained information about treatment 6 months and 1 year after renal biopsy. Conservative therapy was defined as treatment without steroids or immunosuppressants.

### Histopathological examination of PLA2R deposition in renal biopsies

Interpreting the results of PLA2R immunohistochemistry using paraffin-embedded sections can be difficult because the same section may have both positive and negative glomeruli. Moreover, enzyme immunoassay techniques for detection are prone to false positives [15]. For these reasons, glomerular PLA2R deposition was detected in frozen sections using IF.

**Figure 1:**
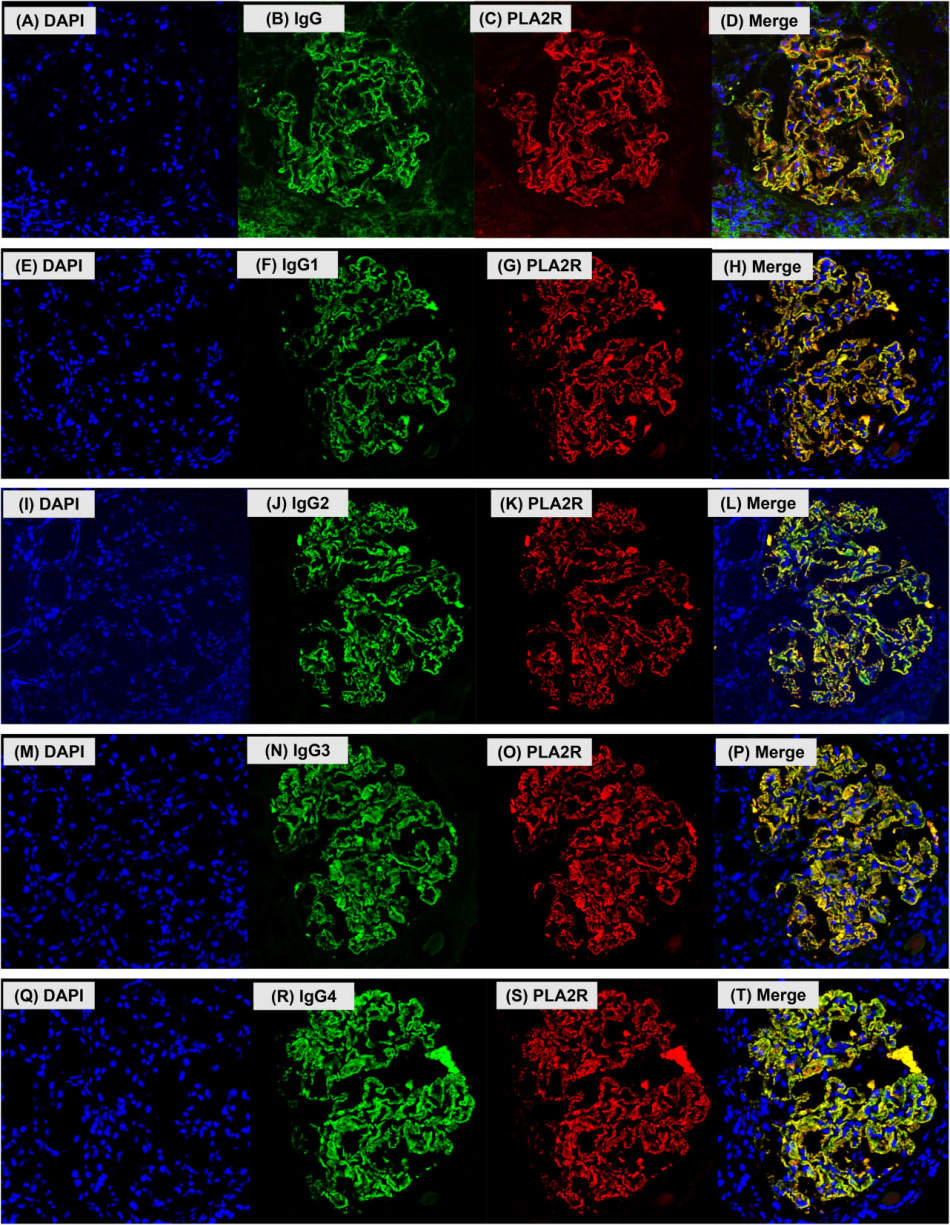
Confocal light microscopy images demonstrating the co-localization of PLA2R with total IgG and specific IgG subclasses. **(A–D)** Images show co-localization of PLA2R and whole IgG: **(A)** DAPI, **(B)** IgG, **(C)** PLA2R, **(D)** Merge. **(E–H)** Images show co-localization of PLA2R and IgG1: **(E)** DAPI, **(F)** IgG1, **(G)** PLA2R, **(H)** Merge. **(I–L)** Images show co-localization of PLA2R and IgG2: **(I)** DAPI, **(J)** IgG2, **(K)** PLA2R, **(L)** Merge. **(M–P)** Images show co-localization of PLA2R and IgG3: **(M)** DAPI, **(N)** IgG3, **(O)** PLA2R, **(P)** Merge. **(Q–T)** Images show co-localization of PLA2R and IgG4: **(Q)** DAPI, **(R)** IgG4, **(S)** PLA2R, **(T)** Merge. DAPI: 4′,6-diamidino-2-phenylindole.

PLA2R is weakly expressed in normal glomeruli. We confirmed the co-localization of PLA2R and IgG in subepithelial immunocomplexes by double-staining with anti-PLA2R and anti-IgG antibodies (Fig. 1A–D). To further demonstrate the co-localization of an IgG subclass with PLA2R, co-staining was performed with the same method.

For immunohistochemistry, thin frozen sections (2 µm) were air dried for 30 minutes and hydrated with phosphate-buffered saline (PBS). Subsequently, non-specific binding was blocked by incubating with Blocking One (Nacalai Tesque, Kyoto, Japan) for 30 minutes. Then the sections were incubated with anti-PLA2R antibody (1:2000; HPA012657, Atlas Antibodies, Stockholm, Sweden) and anti-IgG antibody (used at the stock concentration; A57H, Nichirei Biosciences, Tokyo, Japan) overnight at 4°C. After washing twice with PBS, we incubated the specimens with Alexa Fluor 568 goat anti-rabbit IgG (1:1000; Invitrogen, Carlsbad, CA, USA) and Alexa Fluor 488 goat anti-mouse IgG (1:1000; Invitrogen) for 60 min. After washing twice with PBS, we mounted the slides and counterstained with Vectashield Mounting Medium with DAPI (Vector Laboratories, Burlingame, CA, USA). Microscopic images were acquired with a fluorescence microscope and a Zeiss LSM 700 confocal microscope (Zeiss, Jena, Germany). The intensity of PLA2R deposition was defined as 1+ (distinctly positive), 2+ (strongly positive) and 3+ (strongly positive with saturation). The glomerular PLA2R intensities of all cases were evaluated and imaged by one nephrologist (K.U., observer 1) for the main analysis and two other nephrologists (observers 2 and 3) to evaluate reproducibility using the image data captured by K.U.

### Outcomes

The primary outcome was the rate of complete remission of proteinuria. The secondary outcomes were the development of ESKD and partial remission of proteinuria.

The definition of remission was determined according to previous reports [16]. Two consecutive urinary protein levels <0.3 g protein/g creatinine (g/gCr) was considered indicative of complete remission of proteinuria. Partial remission was defined as 50% proteinuria reduction from the peak level and urinary protein levels <3.5 g/gCr.

### Statistical analysis

Data were expressed as frequency and percentage or median and interquartile range, as appropriate. Categorical variables in the PLA2R-negative and PLA2R-positive groups were compared via Fisher's exact test, and continuous variables were compared via the *t*-test or Mann–Whitney U test. Kaplan–Meier survival analysis with logrank test was performed to compare the remission rates. To analyse the factors associated with a first complete remission of urinary protein, the Cox proportional hazards model was used and hazard ratios were calculated. Adjustment variables in the multivariate analysis were selected as follow: age, sex, UPCR, serum levels of albumin and creatinine, which have previously been reported to be associated with complete remission, as well as the intensity of glomerular PLA2R and IgG subclass staining, which were the factors under investigation in this study. Reproducibility between the findings of K.U. (observer 1) and each nephrologist was assessed with Cohen's weighted κ coefficient, and reproducibility between all three was assessed with Fleiss's κ coefficient. A two-tailed *P*-value of .05 was deemed to indicate statistical significance for all statistical analyses. All data were analysed using JMP 13.0.0 (SAS Institute, Cary, NC, USA).

## RESULTS

### Baseline characteristics and comparison of clinicopathological characteristics

The baseline characteristics of all patients are shown in Table 1. There was a mean age of 66 years among the 132 individuals, 53.0% of whom were male. The median follow-up time after renal biopsy was 78 months. The number (proportion) of glomerular PLA2R-positive patients was 101 (76.5%) and there were 31 (23.5%) negative patients. The percentages of cases positive for each IgG subclass were IgG1, 98.5%; IgG2, 40.2%; IgG3, 38.6% and IgG4, 87.9%. A total of 19 cases had a history of malignancy, including 4 cases of malignant lymphoma, 3 cases of gastric cancer, 2 cases of colorectal cancer, 2 cases of multiple myeloma, 2 cases of pancreatic tumour and 1 case each of submandibular gland cancer, thyroid cancer, brain tumour, breast cancer, lung cancer and bladder cancer. Cases with malignancy onset within 1 year or active malignancies at the time of renal biopsy were excluded from the study. The median UPCR and serum albumin level were 4.9 g/gCr and 2.5 g/dl, respectively. The number (proportion) of individuals who received corticosteroids or immunosuppressant therapy at 6 months and 1 year after renal biopsy was 72 (54.5%) and 75 (56.8%), respectively.

**Table 1:** Baseline characteristics at the time of renal biopsy.

Characteristics	All patients (*N* = 132)	PLA2R negative MN (*n* = 31)	PLA2R positive MN (*n* = 101)	*P*-value
Male, *n* (%)	70 (53.0)	19 (61.3)	51 (50.5)	0.312
Age (years), median (IQR)	66 (56–73)	68 (50–75)	65 (58–73)	0.584
Follow-up (months), median (IQR)	78 (45–125)	59 (26–88)	84 (48–140)	0.016
Findings of renal biopsy, *n* (%)				
PLA2R positivity	101 (76.5)	-	-	-
Glomerular IgG1 positivity	130 (98.5)	29 (93.6)	101 (100)	0.054
Glomerular IgG2 positivity	53 (40.2)	8 (25.8)	45 (44.6)	0.093
Glomerular IgG3 positivity	51 (38.6)	9 (29.0)	42 (41.6)	0.292
Glomerular IgG4 positivity	116 (87.9)	19 (61.3)	97 (96.0)	<0.001
Spike formation	65 (49.2)	12 (38.7)	53 (52.5)	0.220
GBM thickening	84 (63.6)	14 (45.2)	70 (69.3)	0.019
GBM double contour	20 (15.2)	2 (6.5)	18 (17.8)	0.158
IFTA ≥2+	15 (11.4)	3 (9.7)	12 (11.9)	1.000
Interstitial cell infiltration ≥2+	10 (7.6)	1 (3.2)	9 (8.9)	0.451
Clinical findings				
Duration from onset to renal biopsy (days), median (IQR)	83 (34–219)	75 (32.5–248)	85 (34.5–219)	0.409
Past history of malignancy, *n* (%)	19 (14.4)	6 (19.4)	13 (12.9)	0.387
Systolic BP (mmHg), median (IQR)	135 (120–148)	126 (110–142)	137 (122–150)	0.014
Diastolic BP (mmHg), median (IQR)	78 (70–87)	72 (68–84)	80 (71–88)	0.028
Haematuria, *n* (%)	64 (49.2)	12 (40.0)	52 (52.0)	0.300
UPCR (g/gCr), median (IQR)	4.9 (2.7–8.3)	5.6 (2.5–8.4)	4.6 (2.7–8.2)	0.580
Serum total protein (g/dl), median (IQR)	5.4 (4.7–6.0)	5.7 (4.7–6.7)	5.3 (4.7–5.9)	0.131
Serum albumin (g/dl), median (IQR)	2.5 (2.0–3.2)	2.7 (2.0–3.4)	2.5 (2.0–3.1)	0.673
Blood urea nitrogen (mg/dl), median (IQR)	15.0 (11.0–20.0)	15.7 (12.0–23.0)	14.1 (11.0–19.9)	0.475
Serum creatinine (mg/dl), median (IQR)	0.87 (0.64–1.15)	0.90 (0.66–1.20)	0.84 (0.64–1.09)	0.447
Serum total cholesterol (mg/dl), median (IQR)	285 (223–371)	325 (241–400)	269 (217–368)	0.170
Serum triglyceride (mg/dl), median (IQR)	163 (122–225)	197 (122–323)	159 (119–216)	0.089
Serum IgG (mg/dl), median (IQR)	806 (631–1139)	962 (718–1260)	772 (621–1003)	0.090
Treatment 6 months after renal biopsy, *n* (%)				
Only conservative therapy	60 (45.5)	12 (38.7)	48 (47.5)	0.750
Corticosteroids monotherapy	28 (21.2)	6 (19.4)	22 (21.8)	0.771
Corticosteroids and immunosuppressant	41 (31.0)	12 (38.7)	29 (28.7)	0.293
Immunosuppressant monotherapy	3 (2.3)	1 (3.2)	2 (2.0)	0.684
Treatment 1 year after renal biopsy, *n* (%)				
Only conservative therapy	57 (43.2)	12 (38.7)	45 (44.6)	0.680
Corticosteroids monotherapy	29 (22.0)	6 (19.4)	23 (22.8)	0.688
Corticosteroids and immunosuppressants	43 (32.6)	12 (38.7)	31 (30.7)	0.511
Immunosuppressant monotherapy	3 (2.3)	1 (3.2)	2 (2.0)	0.555

The baseline characteristics stratified by glomerular PLA2R-positive or PLA2R-negative MN are also shown in Table 1. Follow-up time, glomerular IgG4 positivity, GBM thickening and BP were all significantly higher in the glomerular PLA2R-positive MN group than in the glomerular PLA2R-negative MN group. With regard to treatment, the rates of corticosteroid and immunosuppressant therapy were not significantly different between the glomerular PLA2R-negative and PLA2R-positive MN groups. The only immunosuppressants used were cyclosporin A or mizoribine. In the glomerular PLA2R-positive MN group, the rates of IgG1 and IgG4 deposition were high (100% and 96.0%, respectively) and the rates of IgG2 and IgG3 deposition were relatively low (44.6% and 41.6%, respectively). Co-localization of every IgG subclass with PLA2R as observed (Fig. 1).

### Renal outcome and remission rate based on PLA2R deposition

**Figure 2:**
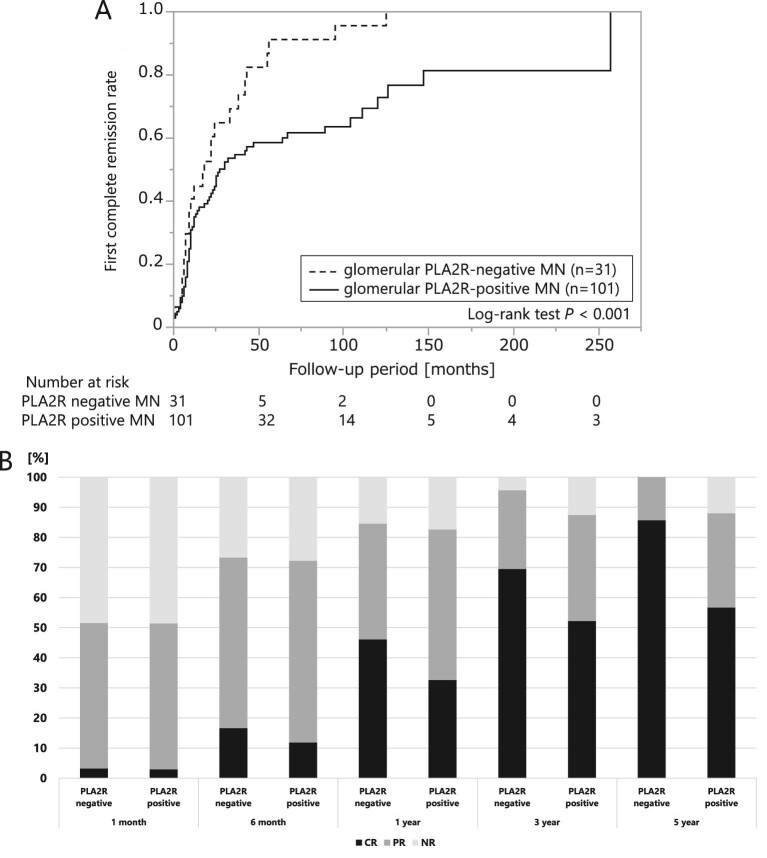
Differences in remission rate according to the presence of glomerular PLA2R staining. **(A)** Kaplan–Meier analysis of the first complete remission rates of the glomerular PLA2R-positive MN group and the glomerular PLA2R-negative MN group. **(B)** The bar chart shows the rates of partial and complete remission at 1 month, 6 months, 1 year, 3 years and 5 years after renal biopsy for the PLA2R-negative and PLA2R-positive MN groups. CR: complete remission; PR: partial remission; NR: no remission.

The number of patients who developed ESKD did not significantly differ between the glomerular PLA2R-negative (*n* = 1) and glomerular PLA2R-positive (*n* = 8) MN groups (data not shown; logrank test *P* = .608). We observed 4 patients among the PLA2R-negative cases and 13 among the PLA2R-positive cases who experienced a doubling of their serum creatinine levels (data not shown; logrank test *P* = .682). The percentages of patients who achieved complete remission over the whole observational period and 1 year after renal biopsy were 68.9% and 34.4%, respectively. During the entire observation period, the glomerular PLA2R-positive MN group had a worse rate of first complete remission than the glomerular PLA2R-negative MN group (Fig. 2A; logrank test *P* < .001). The same was also true for the observation period within 50 months after diagnosis (Supplementary Fig. S2A; logrank test *P* = .032). A stratified analysis of the treated and untreated groups with glucocorticoids or immunosuppressant found no interaction between the two groups (Supplementary Fig. S3A). The rates of partial or complete remission did not differ between the glomerular PLA2R-positive and glomerular PLA2R-negative MN groups (data not shown; logrank test *P* = .943). Fig. 2B shows the numbers of individuals in partial and complete remission at 1 month, 6 months, 1 year, 3 years and 5 years after renal biopsy for the PLA2R-negative and PLA2R-positive MN groups. Comparing PLA2R-positive MN with PLA2R-negative MN cases, there was no difference in the rate of remission 1 month after renal biopsy. However, the number of remission cases in both groups increased with time, eventually revealing a higher remission rate for the group with PLA2R-negative MN, with a significantly higher complete remission rate after 5 years (*P* = .042).

**Figure 3:**
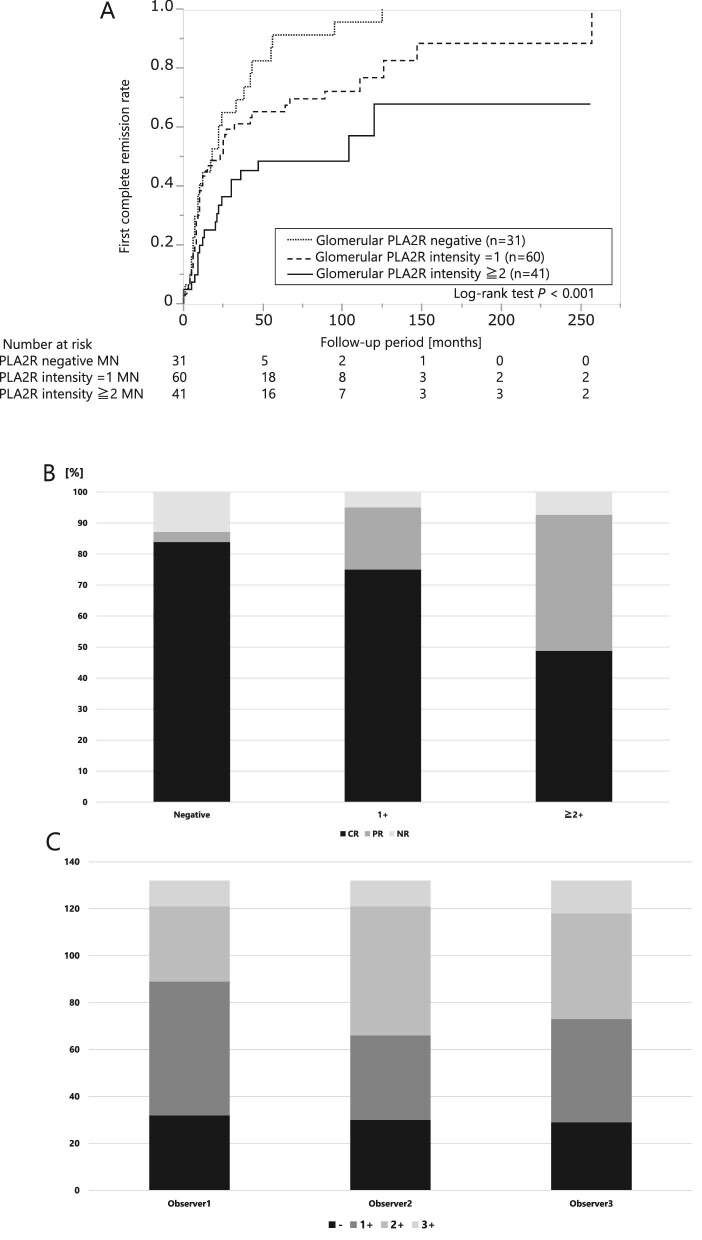
Association between glomerular PLA2R staining intensity and proteinuria remission rate. **(A)** Kaplan–Meier analysis of the first complete remission rates in each group with negative, 1+ and ≥2 glomerular PLA2R intensity. **(B)** The bar chart shows the rates of partial and complete remission for the entire observation period stratified by the intensity of glomerular PLA2R staining. **(C)** The bar chart shows the distribution of the evaluations of PLA2R intensity for each of the three observers in the interobserver reproducibility analysis. CR: complete remission; PR: partial remission; NR: no remission.

The group with a glomerular PLA2R intensity ≥2+ had a poor first complete remission rate compared with the group with low PLA2R intensity during the entire observation period (Fig. 3A; logrank test *P* < .001). Moreover, the complete remission rate for the entire observation period was lower for cases with stronger glomerular PLA2R intensity (Fig. 3B). The results were similar within the 50-month observation period (Supplementary Fig. S2B; logrank test *P* = .020). The Cox proportional hazards analyses revealed that strong glomerular PLA2R staining (≥2+) was significantly associated with a failure to reach complete remission (Table 2). The association remained significant whether adjusted for glomerular IgG subclass staining {model 1, hazard ratio [HR] 1.72 [95% confidence interval (CI) 1.03–2.89], *P* = .037} or for clinical factors [model 2, HR 2.09 (95% CI 1.27–3.47), *P* = .004]. A stratified analysis of the treated and untreated groups with glucocorticoids or immunosuppressants found no interaction between the two groups (Supplementary Fig. S3B). The duration from onset of urinary protein to renal biopsy was not significantly different for glomerular intensities of 1+ and ≥2+, with a median of 89 days and 83 days, respectively (*P* = .531). Fig. 3C shows the distribution of the evaluation of PLA2R intensity for each of the three observers in the interobserver reproducibility analysis. The rate of complete agreement among the three observers was 65%. Fleiss's κ coefficient was 0.672, which indicated substantial agreement. Moreover, agreement between observer 1 and each of the other two observers was 70% and 79% with Cohen's weighted κ coefficients of 0.824 and 0.875, respectively, indicating near-perfect reproducibility.

**Table 2:** Cox proportional hazards model for first complete remission of urinary protein.

Variables	Population at risk	Remissions, *n*	Unadjusted hazard ratio (95% CI)	*P*-value	Age- and sex-adjusted hazard ratio (95% CI)	*P*-value	Multivariate-adjusted hazard ratio (95% CI). model 1	*P*-value	Multivariate-adjusted hazard ratio (95% CI). model 2	*P*-value
All cases (*N* = 132)										
Intensity of glomerular PLA2R staining <2	91	71	1.00	<0.01	1.00	<0.01	1.00	0.037	1.00	0.004
Intensity of glomerular PLA2R staining ≥2	41	20	2.03 (1.23–3.33)		2.06 (1.25–3.40)		1.72 (1.03–2.89)^a^		2.09 (1.27–3.47)^b^	
PLA2-positive MN (*n* = 101)										
Glomerular IgG2 staining (negative)	56	41	1.00	0.033	1.00	0.030	1.00	0.041	1.00	0.030
Glomerular IgG2 staining (positive)	45	24	1.73 (1.04–2.90)		1.77 (1.06–2.97)		1.73 (1.00–3.00)^c^		1.78 (1.06–3.00)^b^	

Model 1 adjusted for glomerular PLA2R and IgG subclass staining, which were the factors under investigation in this study.

Model 2 adjusted for factors previously reported to be associated with complete remission.

a Adjusted for sex, age, glomerular IgG1 staining, glomerular IgG2 staining, glomerular IgG3 staining and glomerular IgG4 staining.

b Adjusted for sex, age, UPCR, serum albumin level and serum creatinine level.

c Adjusted for sex, age, intensity of glomerular PLA2R staining, glomerular IgG1 staining, glomerular IgG3 staining and glomerular IgG4 staining.

### Association between glomerular IgG subclass staining and complete remission rate

**Figure 4:**
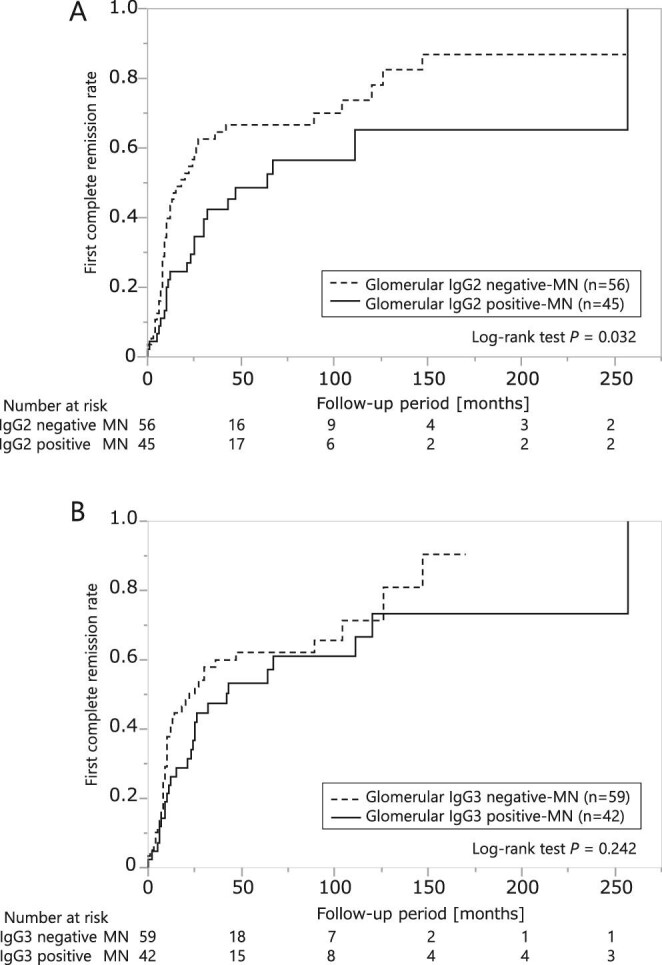
Difference in urinary protein remission rate according to IgG subclass staining in patients with glomerular PLA2R-positive MN. **(A)** In the glomerular PLA2R-positive MN group, Kaplan–Meier analysis of the first complete remission rates of the glomerular IgG2-negative and positive MN groups. **(B)** In the glomerular PLA2R-positive MN group, Kaplan–Meier analysis of the first complete remission rates of the glomerular IgG3-negative and positive MN groups.

We also performed subgroup analyses according to glomerular IgG subclass staining. Fig. 1 shows images featuring the co-localization of PLA2R and the various IgG subclasses (E–H: IgG1, I–L: IgG2, M–P: IgG3 and Q–T: IgG4). Every IgG subclass co-localized with PLA2R. In the glomerular PLA2R-positive MN group, the patients with glomerular IgG2 staining in their renal biopsy had a poorer first complete remission rate than the patients without IgG2 during the entire observation period (Fig. 4A; logrank test *P* = .032). The results were similar within the 50-month observation period (Supplementary Fig. S2C; logrank test *P* = .025). However, glomerular IgG3 staining was not associated with the first complete remission (Fig. 4B; logrank test *P* = .242). The Cox proportional hazards analyses revealed that glomerular IgG2 positivity was a significant risk factor for failing to reach remission (Table 2). The association remained significant whether adjusted for glomerular IgG subclass staining [model 1, HR 1.73 (95% CI 1.00–3.00), *P* = .041] or for clinical factors [model 2, HR 1.78 (95% CI 1.06–3.00), *P* = .030]. A stratified analysis of the treated and untreated groups with glucocorticoids or immunosuppressants found no interaction between the two groups (Supplementary Fig. S3C).

In the glomerular PLA2R-negative MN group, contrary to PLA2R-positive cases, the patients with glomerular IgG3 staining had a poorer first complete remission rate than the patients without IgG3 (data not shown; logrank test *P* = .002), and glomerular IgG2 staining was not associated with the first complete remission (data not shown; logrank test *P* = .988).

## DISCUSSION

This study reveals that glomerular PLA2R and IgG subclass staining in samples from idiopathic MN patients have clinical implications. We revealed that glomerular PLA2R-positive MN cases, particularly those with high-intensity glomerular PLA2R deposition, or glomerular IgG2-positive MN cases have a poor remission rate. These results were significant in multivariate analyses using the Cox proportional hazards model.

We observed PLA2R staining in approximately 77% of samples, which was higher than previously reported in Japan [15, 17] and similar to previous reports from the West and China. Serum anti-PLA2R antibody positivity was lower among patients from Japan than among patients from elsewhere [18], which was attributed to relatively weak MN disease severity. Glomerular PLA2R staining is expected to be more accurate than serum anti-PLA2R antibody analysis for diagnosis because it is positive even when disease is weak, but glomerular staining can be difficult to interpret because even normal glomeruli are weakly positive. We calculated a more accurate positivity rate than in previous reports by co-staining with IgG. Notably, our results suggest that the prevalence of PLA2R-type MN in Japan, as diagnosed by glomerular PLA2R staining, does not greatly differ from the prevalence overseas. This finding may change our view on the prevalence of PLA2R-type MN in Japan, which was believed to be lower than in other countries, likely because of cases going undiagnosed because of negative antibody staining. We speculate that measurement of serum anti-PLA2R antibodies alone may not be sufficient to diagnose PLA2R-positive MN, and that immunostaining for glomerular PLA2R is diagnostically important. Previous reports have indicated that PLA2R staining is less specific (91% versus 99%) but more sensitive (78% versus 68%) than serum anti-PLA2R antibody tests [4], and there is concern about false negative results from serum anti-PLA2R antibody measurement alone. It is also possible that our results reflect regional differences within Japan because the glomerular PLA2R positivity rate is higher in the western part of Japan [15, 17].

A previous report showed that glomerular PLA2R-positive MN displayed therapeutic resistance [19]. In the present study, we also found that the glomerular PLA2R-positive MN group had a worse rate of complete remission than the glomerular PLA2R-negative MN group for the longer follow-up period. In particular, higher intensities of glomerular PLA2R staining were associated with lower remission rates. This finding suggests that PLA2R staining intensity reflects the titres of serum anti-PLA2R antibodies. However, we were unable to verify the relationship between glomerular PLA2R intensity in renal biopsy and serum anti-PLA2R titres because serum samples were not available.

Both glomerular PLA2R-positive and IgG2-positive MN cases had poor complete remission rates. Glomerular PLA2R and IgG2 showed co-localization by IF double-staining (Fig. 1I–L). This suggests that IgG2 may constitute immune complexes in such cases, whereas in a previous report, most anti-PLA2R antibodies were IgG4 [11]. The clinicopathological significance of IgG2 positivity in PLA2R-positive MN should be elucidated. IgG2 has a high affinity for oligosaccharides [20]. Oligosaccharides bind lectins, and PLA2R contains a C-type lectin domain [21]. MN with epitope spreading has a poor clinical prognosis [22]. Because both glomerular PLA2R- and IgG2-positive MN are associated with poor clinical prognosis, they may have epitope spreading that involves the C-type lectin domain. Alternatively, an oligosaccharide-associated lectin complement pathway may be involved. It has been reported that MN with a predominance of the lectin pathway is less likely to achieve remission [23], and IgG2 may be involved via the lectin complement pathway. These hypotheses remain to be tested in future studies, as serum samples were not available in this study.

In PLA2R-negative MN, in contrast with PLA2R-positive MN, the patients with glomerular IgG3 staining had a poorer first complete remission rate, and glomerular IgG2 staining was not associated with the first complete remission. However, PLA2R-negative cases are a heterogeneous population in which the number of cases is small and detailed target antigens have not been investigated, thus we believe that the results should be interpreted with caution.

The current study has numerous strengths. First, this study shows the clinical usefulness of glomerular PLA2R staining in renal biopsies. Although the opportunity to evaluate glomerular PLA2R staining is limited to the time of renal biopsy, it can provide important insights into expected remission rates, in that it can reveal glomerular PLA2R-positive MN, which is associated with a poor remission rate, particularly when the intensity of glomerular PLA2R staining is high. Second, this is the first study to reveal the clinical utility of combined glomerular PLA2R and IgG2 staining. Previous reports have dealt with the whole pattern of IgG subclasses, with few reports separately evaluating an IgG subclass other than IgG4. We believe that the novelty of this report is the demonstration that combined evaluation of glomerular PLA2R and the individual IgG subclass has clinical usefulness. This staining method could improve treatment by providing a more accurate prognosis.

The present study also had limitations. First, there are limitations of statistical strength. This is a retrospective observational study, so there could be several biases. For example, the treatments varied by hospital, which may have affected the results, and outcome comparisons between treatments are particularly difficult because of the differing clinical backgrounds. Moreover, long-term prognosis and hard endpoints are limited in their evaluation due to the small number of cases. Second, the study participants were all members of the Japanese population. MN treatment responsiveness in Japan tends to be better than in other countries [24–26]. Patients with MN in Japan are commonly treated with corticosteroid monotherapy or immunosuppressants such as cyclosporin A or mizoribine in accordance with Japanese clinical guidelines of that time [27], which is not recommended in the KDIGO guidelines. In the KDIGO guidelines, cyclophosphamide is indicated for very-high-risk and high-risk groups and rituximab for medium- and high-risk groups, but those treatments are rarely administered in Japan due to insurance coverage issues and side effects. Third, due to the lack of electron microscopy images, it was not possible to verify the association between PLA2R staining and MN morphological staging at high magnification. The final and most important issue is that the serum samples taken at the time of renal biopsy were not retained for further testing. To investigate the relationship between glomerular PLA2R, the IgG2 subclass of anti-PLA2R antibodies and serum titres of anti-PLA2R antibodies, paired renal biopsy and serum samples will be essential.

In conclusion, we revealed that glomerular PLA2R-positive MN, particularly with high-intensity glomerular PLA2R staining, has a poor remission rate. In addition to glomerular PLA2R positivity, glomerular IgG2 positivity in MN is associated with a worse remission rate than glomerular deposition of other IgG subclasses of anti-PLA2R. The clinical relevance of combined glomerular PLA2R and IgG2 staining in MN samples should be studied in detail to improve the diagnosis and treatment of MN.

## Supplementary Material

sfae104_Supplemental_Files

## Data Availability

All data generated or analysed during this study are included in this article and its supplementary information files.

## References

[1] Caravaca-Fontan F, Fernandez-Juarez GM, Floege J et al. The management of membranous nephropathy-an update. Nephrol Dial Transplant 2022;37:1033–42. 10.1093/ndt/gfab316 34748001

[2] Beck LH Jr, Bonegio RG, Lambeau G et al. M-type phospholipase A2 receptor as target antigen in idiopathic membranous nephropathy. N Engl J Med 2009;361:11–21. 10.1056/NEJMoa0810457 19571279 PMC2762083

[3] Hu SL, Wang D, Gou WJ et al. Diagnostic value of phospholipase A2 receptor in idiopathic membranous nephropathy: a systematic review and meta-analysis. J Nephrol 2014;27:111–6. 10.1007/s40620-014-0042-7 24500886

[4] Dai H, Zhang H, He Y. Diagnostic accuracy of PLA2R autoantibodies and glomerular staining for the differentiation of idiopathic and secondary membranous nephropathy: an updated meta-analysis. Sci Rep 2015;5:8803. 10.1038/srep08803 25740009 PMC4350087

[5] Hofstra JM, Beck LH Jr, Beck DM et al. Anti-phospholipase A2 receptor antibodies correlate with clinical status in idiopathic membranous nephropathy. Clin J Am Soc Nephrol 2011;6:1286–91. 10.2215/CJN.07210810 21474589 PMC3109923

[6] Svobodova B, Honsova E, Ronco P et al. Kidney biopsy is a sensitive tool for retrospective diagnosis of PLA2R-related membranous nephropathy. Nephrol Dial Transplant 2013;28:1839–44. 10.1093/ndt/gfs439 23223223

[7] Hoxha E, Kneissler U, Stege G et al. Enhanced expression of the M-type phospholipase A2 receptor in glomeruli correlates with serum receptor antibodies in primary membranous nephropathy. Kidney Int 2012;82:797–804. 10.1038/ki.2012.209 22673885

[8] Doi T, Mayumi M, Kanatsu K et al. Distribution of IgG subclasses in membranous nephropathy. Clin Exp Immunol 1984;58:57–62. 6383668 PMC1576972

[9] Imai H, Hamai K, Komatsuda A et al. IgG subclasses in patients with membranoproliferative glomerulonephritis, membranous nephropathy, and lupus nephritis. Kidney Int 1997;51:270–6. 10.1038/ki.1997.32 8995742

[10] Kuroki A, Shibata T, Honda H et al. Glomerular and serum IgG subclasses in diffuse proliferative lupus nephritis, membranous lupus nephritis, and idiopathic membranous nephropathy. Intern Med 2002;41:936–42. 10.2169/internalmedicine.41.936 12487163

[11] Hofstra JM, Debiec H, Short CD et al. Antiphospholipase A2 receptor antibody titer and subclass in idiopathic membranous nephropathy. J Am Soc Nephrol 2012;23:1735–43. 10.1681/ASN.2012030242 22956816 PMC3458465

[12] Lefaucheur C, Stengel B, Nochy D et al. Membranous nephropathy and cancer: epidemiologic evidence and determinants of high-risk cancer association. Kidney Int 2006;70:1510–7. 10.1038/sj.ki.5001790 16941021

[13] Tanaka S, Ninomiya T, Fujisaki K et al. The Fukuoka Kidney Disease Registry (FKR) Study: design and methods. Clin Exp Nephrol 2017;21:465–73. 10.1007/s10157-016-1294-4 27339444

[14] Kidney Disease: Improving Global Outcomes Glomerular Diseases Work Group . KDIGO 2021 clinical practice guideline for the management of glomerular diseases. Kidney Int 2021;100(4S):S1–276. 34556256 10.1016/j.kint.2021.05.021

[15] Hara S, Goto S, Kamiura N et al. Reappraisal of PLA2R1 in membranous nephropathy: immunostaining method influence and association with IgG4-dominant phenotype. Virchows Arch 2015;467:87–94. 10.1007/s00428-015-1754-3 25820371

[16] Jha V, Ganguli A, Saha TK et al. A randomized, controlled trial of steroids and cyclophosphamide in adults with nephrotic syndrome caused by idiopathic membranous nephropathy. J Am Soc Nephrol 2007;18:1899–904. 10.1681/ASN.2007020166 17494881

[17] Iwakura T, Ohashi N, Kato A et al. Prevalence of enhanced granular expression of thrombospondin type-1 domain-containing 7A in the glomeruli of Japanese patients with idiopathic membranous nephropathy. PLoS One 2015;10:e0138841. 10.1371/journal.pone.0138841 26393352 PMC4578926

[18] Akiyama S, Akiyama M, Imai E et al. Prevalence of anti-phospholipase A2 receptor antibodies in Japanese patients with membranous nephropathy. Clin Exp Nephrol 2015;19:653–60. 10.1007/s10157-014-1054-2 25412738 PMC4543411

[19] Xie Z, Dong W, Li Z et al. Clinical value of renal phospholipase A2 receptor deposit in the prognosis evaluation and treatment options of idiopathic membranous nephropathy: a retrospective cohort study. Nephrology (Carlton) 2020;25:219–29. 10.1111/nep.13691 31900967 PMC7065056

[20] Vidarsson G, Dekkers G, Rispens T. IgG subclasses and allotypes: from structure to effector functions. Front Immunol 2014;5:520. 10.3389/fimmu.2014.00520 25368619 PMC4202688

[21] Ancian P, Lambeau G, Mattei MG et al. The human 180-kDa receptor for secretory phospholipases A2. Molecular cloning, identification of a secreted soluble form, expression, and chromosomal localization. J Biol Chem 1995;270:8963–70. 10.1074/jbc.270.15.8963 7721806

[22] Seitz-Polski B, Dolla G, Payre C et al. Epitope spreading of autoantibody response to PLA2R associates with poor prognosis in membranous nephropathy. J Am Soc Nephrol 2016;27:1517–33. 10.1681/ASN.2014111061 26567246 PMC4849812

[23] Hayashi N, Okada K, Matsui Y et al. Glomerular mannose-binding lectin deposition in intrinsic antigen-related membranous nephropathy. Nephrol Dial Transplant 2018;33:832–40. 10.1093/ndt/gfx235 28992353

[24] Shiiki H, Saito T, Nishitani Y et al. Prognosis and risk factors for idiopathic membranous nephropathy with nephrotic syndrome in Japan. Kidney Int 2004;65:1400–7. 10.1111/j.1523-1755.2004.00518.x 15086481

[25] Yokoyama H, Taguchi T, Sugiyama H et al. Membranous nephropathy in Japan: analysis of the Japan Renal Biopsy Registry (J-RBR). Clin Exp Nephrol 2012;16:557–63. 10.1007/s10157-012-0593-7 22358611

[26] Yamamoto R, Imai E, Maruyama S et al. Incidence of remission and relapse of proteinuria, end-stage kidney disease, mortality, and major outcomes in primary nephrotic syndrome: the Japan Nephrotic Syndrome Cohort Study (JNSCS). Clin Exp Nephrol 2020;24:526–40. 10.1007/s10157-020-01864-1 32146646 PMC7248042

[27] Japanese Society of Nephrology . Guidelines for the treatment of nephrotic syndrome. Nihon Jinzo Gakkai Shi 2011;53:78–122. 21516692

