# Supplementary material for: Combined evaluation of glomerular phospholipase A2 receptor and immunoglobulin G subclass in membranous nephropathy
Source: Clin Kidney J. 2024 Apr 17;17(6):sfae104. doi: 10.1093/ckj/sfae104 (PMC11161704; doi:10.1093/ckj/sfae104)

Suppl Figure 1

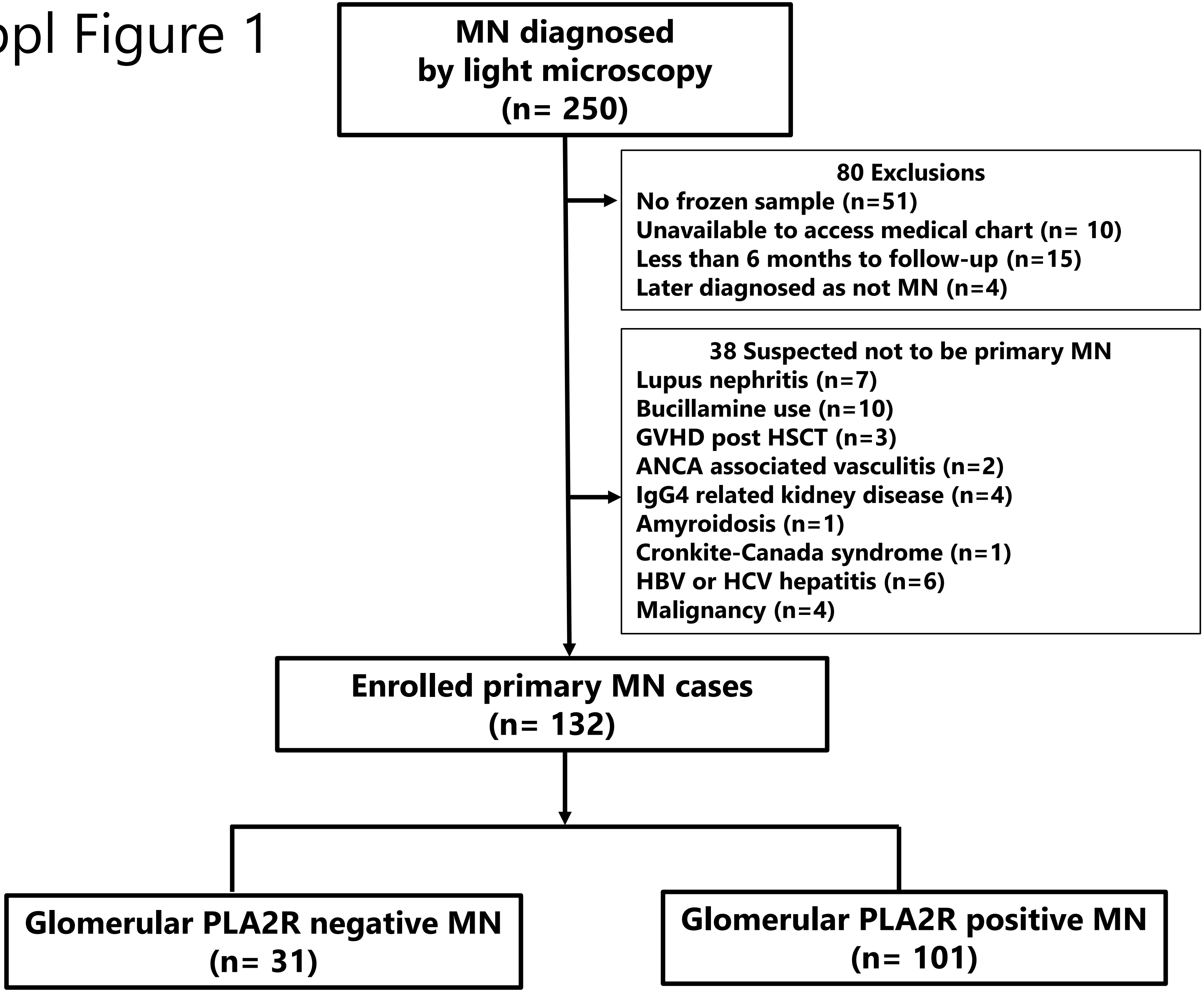

Suppl Figure 2A

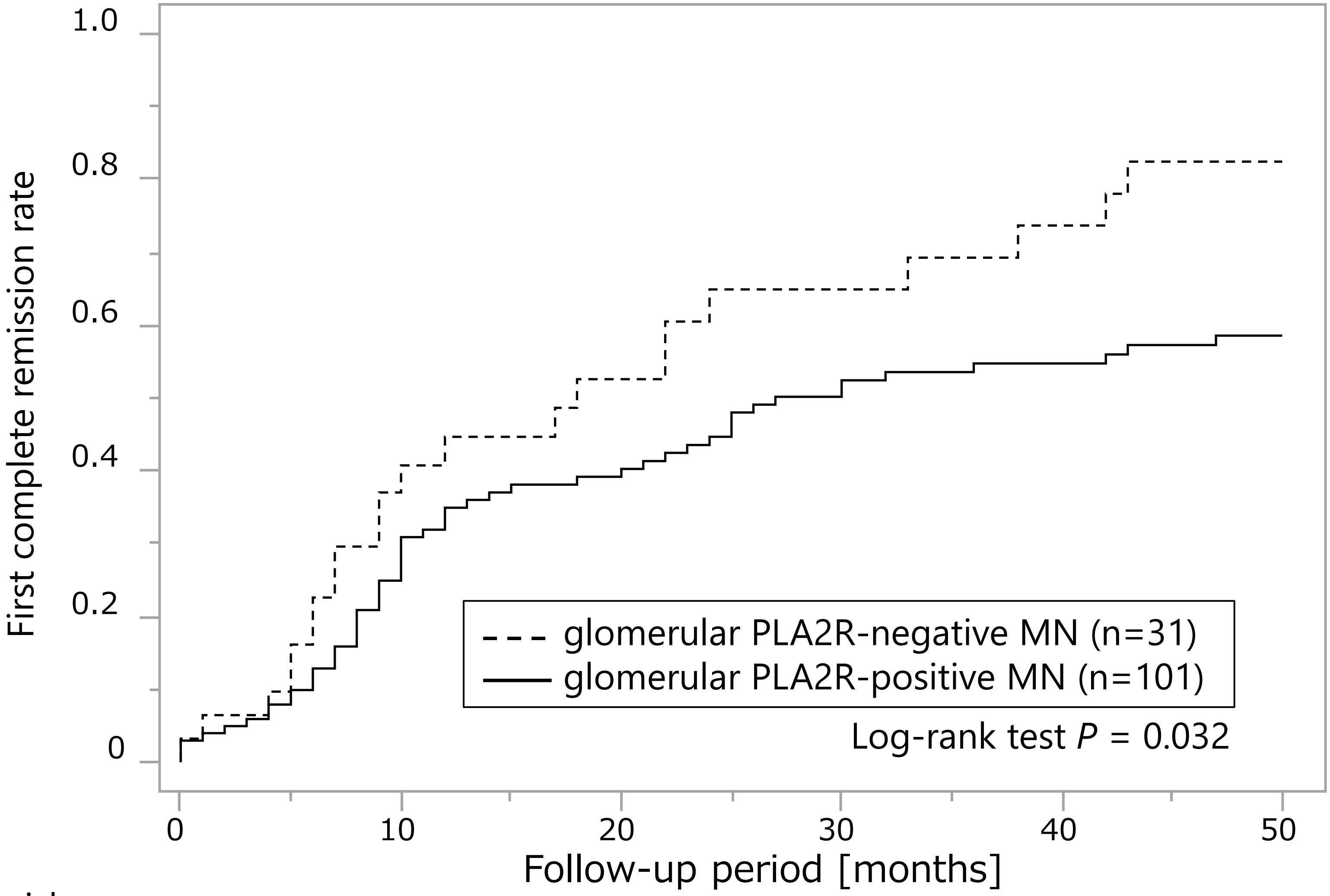

Number at risk						
PLA2R negative MN	31	17	13	9	7	4
PLA2R positive MN	101	75	56	45	37	31

Suppl Figure 2B

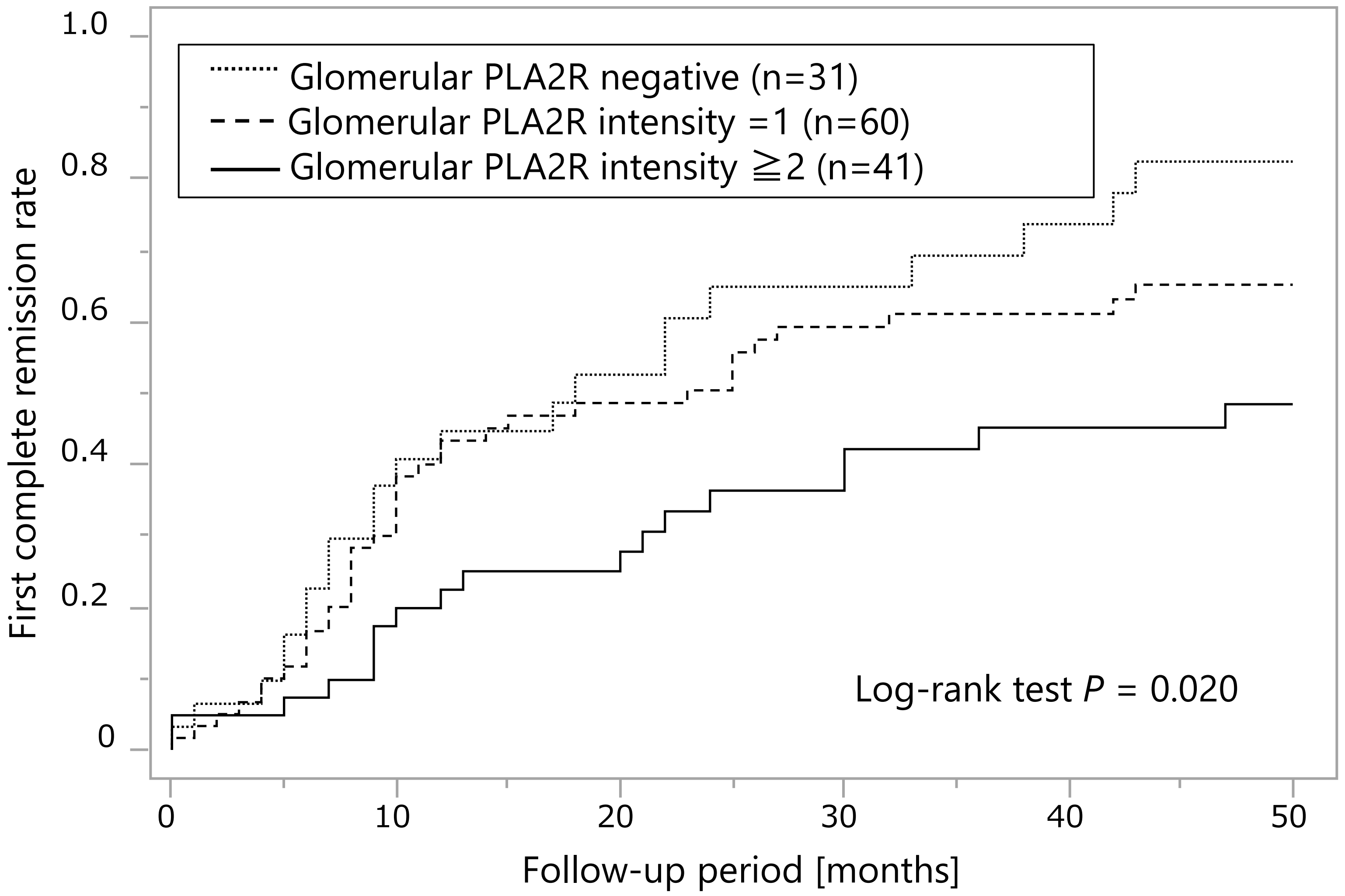

Number at risk						
PLA2R negative MN	31	17	13	9	7	4
PLA2R intensity =1 MN	60	42	30	23	20	16
PLA2R intensity ≧2 MN	41	33	27	22	18	15

Suppl Figure 2C

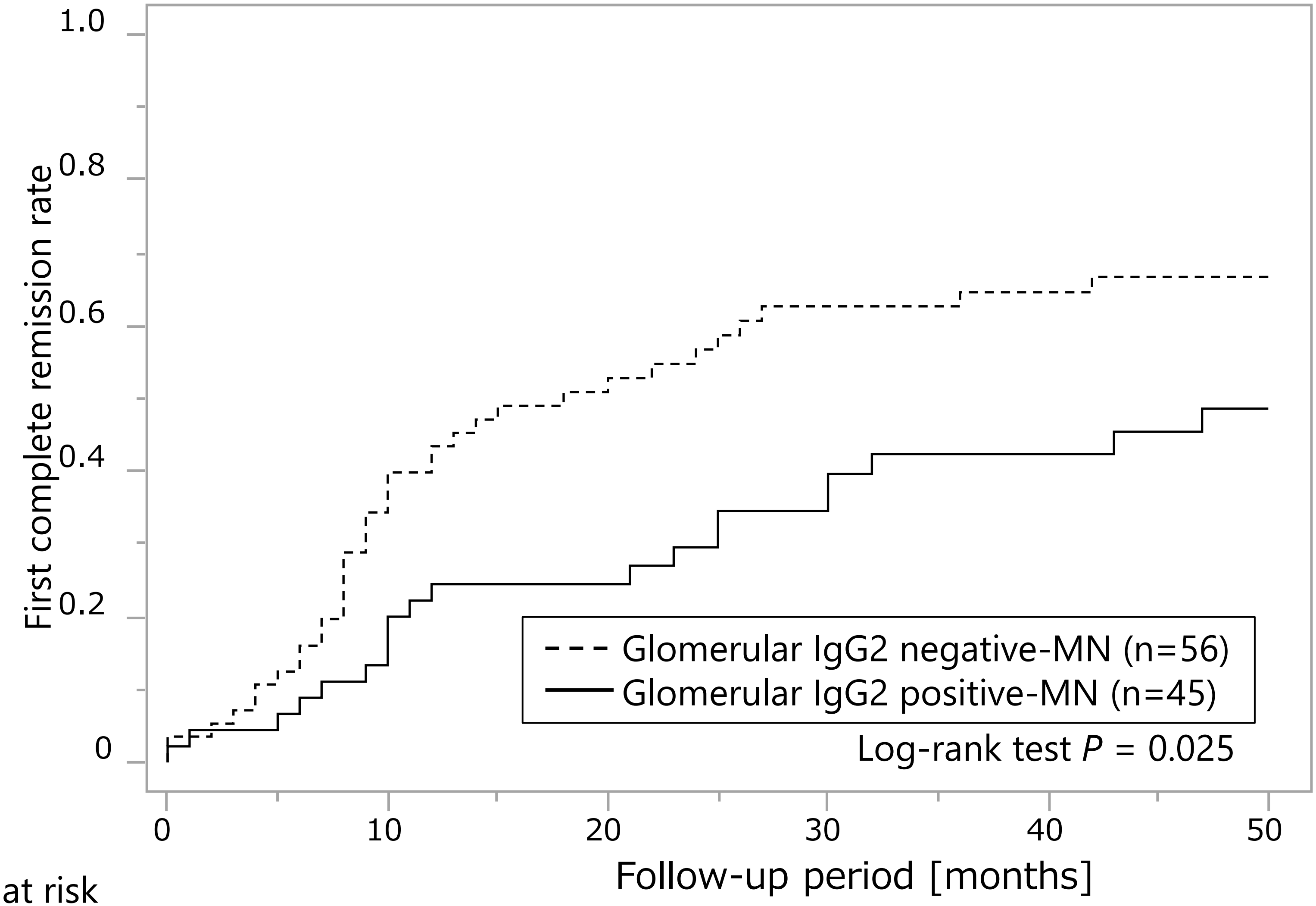

Number at risk

IgG2 negative MN	56	36	26	20	18	15
IgG2 positive MN	45	39	31	26	20	16

Suppl Figure 3A

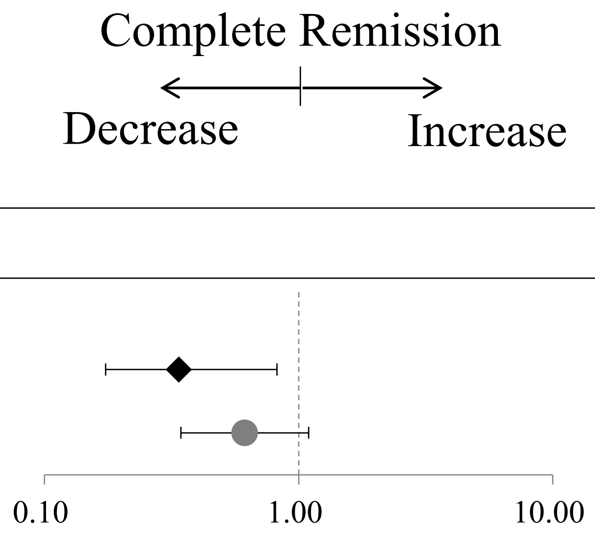

	No. of CR	No. of cases	HR (95%CI)	P for interaction
GC or IS treatment				
Absence	30	50	0.34 (0.17-0.82)	0.397
Presence	61	82	0.61 (0.34-1.09)	

Suppl Figure 3B

Complete Remission

Decrease ⟵|⟶ Increase

	No. of CR	No. of cases	HR (95%CI)	P for interaction
GC or IS treatment				
Absence	20	39	0.40 (0.16-1.00)	0.307
Presence	45	62	0.73 (0.40-1.36)	

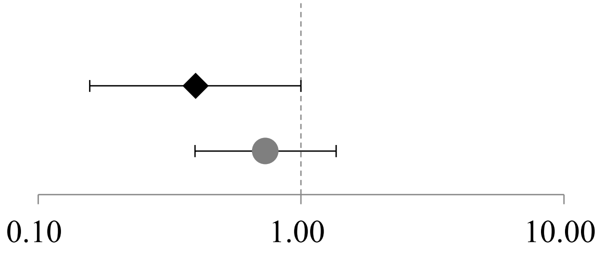

Suppl Figure 3C

Complete Remission

Decrease ⟵ ⟶ Increase

	No. of CR	No. of cases	HR (95%CI)	P for interaction
GC or IS treatment				
Absence	20	39	0.50 (0.17-1.51)	0.953
Presence	45	62	0.51 (0.28-0.95)	

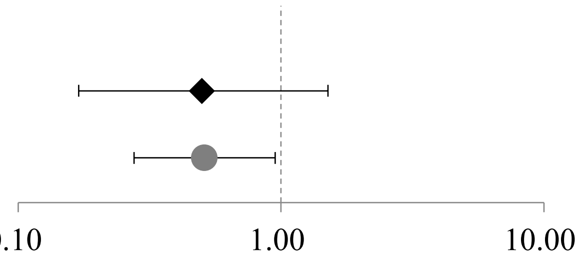

Supplement: sfae104_Supplemental_Files [file sfae104_supplemental_files.zip › SupplFigure.pdf]
